# Wideband excitation of Fano resonances and induced transparency by coherent interactions between Brillouin resonances

**DOI:** 10.1038/s41598-018-27444-8

**Published:** 2018-06-15

**Authors:** Ravi Pant, Siva Shakthi A., Anjali B. Yelikar

**Affiliations:** Laboratory for Phoxonics and Nonlinear Optics in Nanostructures (PHONON), School of Physics, Indian Institute of Science Education and Research (IISER), Thiruvananthapuram, 695551 Kerala India

## Abstract

Wideband excitation and control of Fano resonance and electromagnetically induced transparency (EIT), both of which rely on coherent interaction between two excitation paths, is challenging. It requires precise control and tuning of interacting resonances or coupling between different resonant structures over a wide frequency range. Gain (Stokes) and absorption (anti-Stokes) resonances associated with the stimulated Brillouin scattering (SBS) process can be excited and controlled over a wide frequency range by tuning the pump frequency, its power and profile. We exploit coherent interaction between the Brillouin Stokes and anti-Stokes resonance, in radio frequency domain, to demonstrate Fano and EIT-like resonance over a wide frequency range and control their shape and strength optically and electrically. For the Fano resonance, the asymmetry and polarity are electrically controlled over an unprecedented frequency range (100 MHz–43 GHz) by varying the bias to the intensity modulator whereas, the strength is varied by tuning the Brillouin pump power and/or the bias. The depth and 3 dB linewidth of the transparency window in the EIT-like resonance are controlled using pump and probe parameters. The flexibility of the SBS process that allows wideband electrical and optical control of Fano and EIT-like resonance opens up the potential for applications that range from low-power switching, sensing to tunable RF delay.

## Introduction

Asymmetric shape resonances were observed and studied by Fano for the first time in atomic systems owing to auto-ionization processes^1^. The origin of such asymmetry is devoted to the quantum interference of a discrete (resonant state) with continuum (non-resonant). Since the criterion for the occurrence of Fano resonance involves coherent interaction between two excitation paths, the phenomenon is considered quite generic. Till date, Fano resonances have been observed in many complex dynamical systems such as atomic-plasmonic coupling system^2^; mechanical excitations in nano-optomechanical structures^3,4^; biased semiconductor super lattices^5^; Fano effect in quantum dots^6,7^; Weyl fermions^8^; waveguide-cavity systems^9–11^; perovskites^12^; optically driven atomic force microscopy^13^; and forward stimulated Brillouin scattering (FSBS)^14–17^. Fano resonance finds tremendous applications in microwave spectroscopy of high absorption matter^18,19^; bio-sensors^20^; ultrafast switching^21^; and Fano laser^22,23^. Electromagnetic induced transparency (EIT), on the other hand, arises from the destructive quantum interference between two transition amplitudes and manifests itself as a transparency in an absorption profile^24^. Like Fano resonance, EIT-like resonance has been demonstrated in optomechanical systems^25,26^, photonic crystals^27^, Tera-hertz metamaterials^28^, plasmonic metamaterials^29^, microresonators using FSBS^30,31^, and coupled resonators and microcavities^32,33^. However, *controlled wideband* excitation of both the phenomenon, which is critical for enabling a number of above-mentioned applications, is challenging due to the requirement of precise control of structural or atomic resonance over a large frequency range. It is, therefore, important to harness these phenomena in systems where the interacting pathways can be easily excited and controlled over a wide frequency range.

Stimulated Brillouin scattering (SBS), which occurs as a result of coherent interaction between two optical waves and an acoustic wave, offers tremendous flexibility in terms of wavelength/frequency of operation, bandwidth and spectral profile of the gain and loss resonances associated with it. Depending on the nature of the acoustic mode involved, SBS can be excited using two counter-propagating optical waves, in which case it is termed as backward SBS (BSBS) or using two co-propagating optical waves where it is known as (forward) SBS (FSBS). SBS is also known to be the strongest nonlinearity, which is nearly 100 times stronger than Raman scattering in optical fibers. The flexible nature of the Brillouin process and its high efficiency has made SBS the natural choice for a large number of applications that range from slow-light^31,34–36^, sensing^37^, Brillouin cooling^38^, Brillouin lasers^39,40^ to wideband microwave photonic signal processing^41–45^. These applications are enabled using backward or forward SBS process in optical fibers, photonic chips, silicon nanostructures and waveguides, hybrid circuits and optical resonators. In most of these applications, either a Stokes wave (*ω*_*s*_ = *ω*_*p*_ − Ω_*B*_) or an anti-Stokes wave (*ω*_*as*_ = *ω*_*p*_ + Ω_*B*_) interacts with a Brillouin pump (*ω*_*p*_) to generate an acoustic wave (Ω_*B*_). The observed radio frequency (RF) response in these cases, therefore, has a Lorentzian profile associated with a gain or loss resonance. However, simultaneous excitation of the Stokes (gain) and anti-Stokes (loss) resonance and its effect has remained largely unexplored because anti-Stokes wave undergoes strong absorption and therefore does not generate significant output signal. While the amplitude of output anti-Stokes signal is negligible compared to the Stokes wave, which sees gain, its phase response is of the same order as that of Stokes and result in coherent interaction between the Stokes and anti-Stokes wave whenever their amplitudes are nearly equal.

Here we demonstrate controlled wideband excitation of Fano and EIT-like resonances exploiting the coherent interaction between the Stokes and the anti-Stokes resonance of the BSBS process in the microwave domain. We control the profile, strength, and polarity of the Fano resonance using optical and electrical means over an unprecedented frequency range that extends from 100 MHz to 43 GHz. Our novel approach enables control of the 3 dB linewidth of EIT-like resonance while maintaining the transparency depth. Here we demonstrate that the linewidth can be tuned from 14 MHz to 20 MHz with a fixed transparency depth of 45 dB. Similarly the depth can be tuned with a fixed 3 dB linewidth. For a fixed linewidth of ∼14 MHz, we show that the depth changes from 25 dB to 45 dB for a 4 dB increase in gain. Dynamic control of the Fano resonance and induced transparency over a wide frequency range, using optical and electrical means, opens way for a number of applications in the area of low-power switching, sensing, and microwave photonic signal processing.

## Results

### Excitation of Fano resonance in RF domain

**Figure 1:**
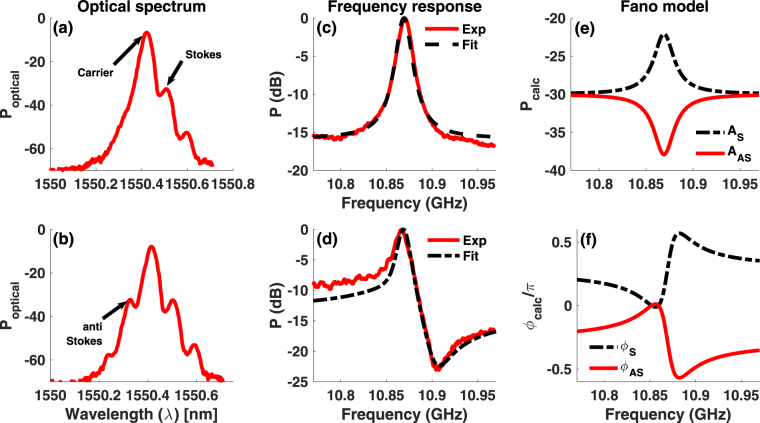
Concept of Fano resonance based on coherent interaction of Stokes and anti-Stokes in RF domain. (**a**) Optical spectrum showing carrier and Stokes signal, obtained by filtering out anti-Stokes sideband. (**b**) Optical spectrum of the probe as filter is tuned out of the anti-Stokes sideband. (**c**) Measured radio frequency response corresponding to optical spectrum in (**a**) showing the characteristic Lorentzian line shape associated with Brillouin gain and simulation fit (dashed). (**d**) Measured RF response (solid) showing the asymmetric Fano-like resonance for the optical signal in (**b**) and calculated Fano spectrum (dash-dot) using Eq. 6. (**e**,**f**) Calculated amplitude and phase spectrum, respectively, for the Stokes (dashed) and anti-Stokes (solid) wave corresponding to the simulation fit in (**d**).

For an optical signal with electric field *E(t)*, radio frequency response is given as: ${S}_{RF}(\omega )=|\int |E(t){|}^{2}{e}^{j\omega t}dt{|}^{2}$ ^46^. In the BSBS process, Stokes (*ω*_*c*_ − Ω_*B*_) and anti-Stokes (*ω*_*c*_ + Ω_*B*_) probe signals are generated by modulating a carrier (*ω*_*c*_) with an RF signal at the Brillouin frequency (Ω_*B*_). By filtering out the anti-Stokes field along the probe arm, Stokes and carrier signals (Fig. 1(a)) are let to counter-propagate with a pump signal of frequency *ω*_*p*_ = *ω*_*c*_. The amplitude and phase of the Stokes signal are affected by the Brillouin gain resonance. When this probe is detected using a wideband photo-detector followed by a vector network analyser (VNA), the resulting RF response at the Brillouin shift arises from the beating of the carrier and the Stokes signal ${S}_{RF}(\omega )=|\int {E}_{c}(t){E}_{s}^{\ast }(t){e}^{j\omega t}dt{|}^{2}$, where *E*_*c*_(*t*) and *E*_*s*_(*t*) are the carrier and Stokes field respectively. The RF spectrum at ${\nu }_{B}=\frac{{{\rm{\Omega }}}_{B}}{2\pi }$, therefore, has the Lorentzian lineshape (Fig. 1(c)), which is characteristic of the Brillouin gain resonance. However, as the filter is tuned away from the anti-Stokes resonance, the probe consists of Stokes, carrier, and anti-Stokes wave (Fig. 1(b)). When the probe in Fig. 1(b) is counter-propagated with the pump, the observed RF signal at *ν*_*B*_ exhibits a Fano-like asymmetry (Fig. 1(d)). The measured RF response at Ω_*B*_ now has two contributions: (i) beat signal between the carrier and Stokes wave and (ii) beat signal between the carrier and anti-Stokes wave and the net beat signal is given by ${S}_{RF}(\omega )=|\int [{E}_{c}(t){E}_{s}^{\ast }(t)+{E}_{c}(t){E}_{as}^{\ast }(t)]{e}^{j\omega t}dt{|}^{2}$ where *E*_*as*_(*t*) is the anti-Stokes signal. Since the carrier is common to the individual beat signals, the RF spectral response results from the coherent interaction between the Brillouin Stokes and anti-Stokes signals.

To generate the Stokes and anti-Stokes sidebands, we use an off-the-shelf single drive Z-cut intensity modulator (Thorlabs), where the electrode is closer to one of the arms of the Mach-Zehnder modulator (MZM). In this case, the upper and lower modulation sidebands see a constant bias dependent phase^47^, which causes a shift in the phase response associated with the Stokes and anti-Stokes resonance (Fig. 1(f)) while keeping the amplitude response unchanged (Fig. 1(e)). By tuning this phase using the bias voltage, condition of destructive interference can be achieved on one side of the resonance. This is at a frequency where the amplitude of the Stokes and anti-Stokes resonance are nearly equal and phase difference is ∼*π* (Fig. 1(e,f)). On the other side of the resonance, the phase difference is much less than *π*, which results in an asymmetric Fano-like resonance. The probe comprising Stokes, anti-Stokes and carrier signals is launched into a 50 m long polarization maintaining (PM) fiber, which is used as the Brillouin medium. To observe Fano resonance, a probe with equal amplitude Stokes and anti-Stokes sidebands is used. Carrier with a larger amplitude is used to generate a large beat signal.

### Optical and electrical control of Fano resonance

**Figure 2:**
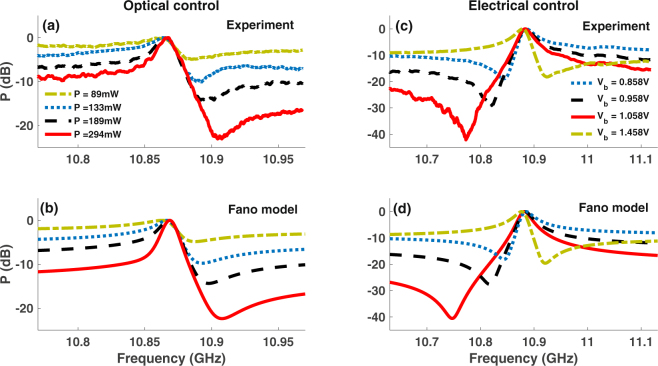
Optical and electrical control of Fano resonance. (**a**) Measured Fano resonance at the Brillouin frequency for different values of the Brillouin pump power while keeping the bias voltage to the probe intensity modulator fixed and (**b**) simulation fit obtained using Eq. 6 with fixed value of bias dependence parameter *α* for different gain values. (**c**) Measured Fano profile showing variation in extinction and frequency of the minimum amplitude point as the bias voltage is tuned at fixed Brillouin gain value. Switching of the Fano profile polarity from having left-handed minimum to right-handed minimum is observed as the bias is tuned from 1.058 V to 1.458 V. (**d**) Simulation fit using different values of *α* parameter in Eq. 6 at a fixed gain value.

To demonstrate optical control of the Fano profile, we vary the Brillouin pump power at a fixed bias voltage. For a fixed probe signal, the Fano resonance becomes stronger as the pump power is increased (Fig. 2(a)) and the frequency, *ν*_*min*_, at which the minima occurs increases. The increase in extinction and *ν*_*min*_ with pump power occurs because it affects both the phase and amplitude of the Stokes and anti-Stokes resonances, which optimizes the condition for destructive interference. Figure 2(b) shows the simulation results from Fano model in Eq. 6 for different values of the gain parameter while keeping the value of the bias dependent *α* parameter fixed.

Electrical control was achieved by varying the voltage bias to the intensity modulator (IM) at a fixed pump power, equivalent to a Brillouin gain of 2.3 dB. Figure 2(c) and (d) show measured and simulation results, respectively, for the control of depth and polarity of the Fano resonance as the bias is changed. Varying the bias affects the phase, which changes the depth and frequency of minima in the Fano profile. The polarity switching corresponds to a change in the sign of parameter *α* in Eq. 6 with bias voltage^48^. The electrical control of Fano resonance has the potential for low power, ultra-fast switching as it changes the RF amplitude at a given frequency by 30 dB for a small change in applied bias voltage ∼0.2 V (solid and dots) (Fig. 2(c,d)). From Fig. 2(a–d), it is evident that the profile, polarity, and extinction, defined as the ratio of maximum to minimum amplitude, of the Fano resonance can be controlled via optically and electrically induced phase shifts.

In order to model the coherent interaction between the Stokes and anti-Stokes resonances, we assume that the optical field emerging from a Z-cut intensity modulator is given according to^48^:

1 $$E(t)=\frac{{E}_{o}}{2}{e}^{j{\omega }_{c}t}[{e}^{j{\varphi }_{01}}{e}^{j{m}_{1}\cos {\omega }_{m}t}+{e}^{j{\varphi }_{02}}{e}^{j{m}_{2}\cos {\omega }_{m}t}]$$

where *ω*_*c*_, and *ϕ*_01,02_ denote laser (carrier) frequency and bias induced phase shifts, respectively, in two arms of the MZM. The modulation frequency and amplitude for each arm are represented by *ω*_*m*_, *m*_1_ and *m*_2_. For small signal modulation, the output of the MZM can be written as^47^:

2 $$E(t)=\frac{{E}_{o}}{2}{e}^{j{\omega }_{c}t}{e}^{j{\varphi }_{0}}\mathrm{[2}\,\cos \,{\rm{\Delta }}{\varphi }_{0}+m\,\sin \,{\rm{\Delta }}{\varphi }_{0}(1+j\alpha )({e}^{j{\omega }_{m}t}+{e}^{-j{\omega }_{m}t})].$$

Here $${\varphi }_{0}=\frac{{\varphi }_{01}+{\varphi }_{02}}{2}$$, $${\rm{\Delta }}{\varphi }_{0}=\frac{{\varphi }_{01}-{\varphi }_{02}}{2}$$ and *m* = *m*_2_ − *m*_1_.

We define a parameter *α* that depends on ${\rm{\Delta }}{\varphi }_{0}$, *m*_1_ and *m*_2_ as follows:

3 $$\alpha =-\,\frac{{m}_{1}+{m}_{2}}{{m}_{1}-{m}_{2}}\,\cot ({\rm{\Delta }}{\varphi }_{0}).$$

When the modulated signal is propagated through a Brillouin active medium, the amplitude and phase of the upper and lower sidebands are modified by Brillouin loss and gain, respectively, when *ω*_*m*_ is scanned around Ω_*B*_. The resulting output probe field is then given as^41^:

4 $$\begin{array}{rcl}{E}_{out}(t) & = & \frac{{E}_{o}}{2}{e}^{j{\omega }_{c}t}{e}^{j{\varphi }_{0}}\mathrm{\{2}\,\cos \,{\rm{\Delta }}{\varphi }_{0}\\  &  & +\frac{m\,\sin \,{\rm{\Delta }}{\varphi }_{0}}{2}\sqrt{1+{\alpha }^{2}}[|{G}_{as}({\omega }_{c}+{\omega }_{m})|{e}^{j[{\omega }_{m}t+{\tan }^{-1}\alpha -{\varphi }_{as}({\omega }_{c}+{\omega }_{m})]}\\  &  & +|{G}_{s}({\omega }_{c}-{\omega }_{m})|{e}^{-j[{\omega }_{m}t-{tan}^{-1}\alpha +{\varphi }_{s}({\omega }_{c}-{\omega }_{m})]}]\}.\end{array}$$

where

$$|{G}_{s}({\omega }_{c}-{\omega }_{m})|={e}^{\frac{G\mathrm{/2}}{1+4{(\frac{{\omega }_{m}-{{\rm{\Omega }}}_{B}}{{{\rm{\Gamma }}}_{b}})}^{2}}}$$

$$|{G}_{as}({\omega }_{c}+{\omega }_{m})|={e}^{-\frac{G\mathrm{/2}}{1+4{(\frac{{\omega }_{m}-{{\rm{\Omega }}}_{B}}{{{\rm{\Gamma }}}_{b}})}^{2}}}$$

$${\varphi }_{s}({\omega }_{c}-{\omega }_{m})={\varphi }_{as}({\omega }_{c}+{\omega }_{m})=\frac{G(\frac{{\omega }_{m}-{{\rm{\Omega }}}_{B}}{{{\rm{\Gamma }}}_{b}})}{1+4{(\frac{{\omega }_{m}-{{\rm{\Omega }}}_{B}}{{{\rm{\Gamma }}}_{b}})}^{2}}$$

Detection of the probe field using a broadband detector gives the signal at frequency *ω*_*m*_, which results from the beating of the carrier with Stokes and anti-Stokes and is calculated according to:

5 $$\begin{array}{rcl}I({\omega }_{m}) & = & \frac{m{I}_{o}}{4}\sqrt{1+{\alpha }^{2}}\,\sin (2{\rm{\Delta }}{\varphi }_{0})\\  &  & [|{G}_{as}({\omega }_{c}+{\omega }_{m})|\,\cos ({\omega }_{m}t+{\tan }^{-1}\alpha -{\varphi }_{as}({\omega }_{c}+{\omega }_{m}))\\  &  & +|{G}_{s}({\omega }_{c}-{\omega }_{m})|\,\cos ({\omega }_{m}t-{\tan }^{-1}\alpha +{\varphi }_{s}({\omega }_{c}-{\omega }_{m}))].\end{array}$$

The RF power at frequency *ω*_*m*_ can then be obtained using:

6 $${S}_{RF}=\frac{{m}^{2}{I}_{0}^{2}\mathrm{(1}+{\alpha }^{2}){\sin }^{2}(2{\rm{\Delta }}{\varphi }_{0})}{16}{ {\mathcal R} }^{2}R[|{G}_{as}{|}^{2}+|{G}_{s}{|}^{2}+\mathrm{2|}{G}_{as}||{G}_{s}|\,\cos (2{\tan }^{-1}\alpha -{\varphi }_{s}-{\varphi }_{as})],$$

where ${\mathcal R}$ and *R* are detector responsivity and load resistance. From Eqs (5) and (6), it is evident that the Fano resonance at *ω*_*m*_ results from the coherent interaction between the Stokes and anti-Stokes resonance. Since the amplitude and phase of the Stokes and anti-Stokes excitation depend on the Brillouin pump power, Fano resonance profile can be controlled optically by varying the pump power. The dependence of the phase of the two coherently interacting pathways on intensity modulator bias voltage, through parameter *α*, enables electrical control. We use our model to obtain a fit to the Fano resonance in Figs 1 and 2 to understand the origin of the extinction and polarity switching. The amplitude and phase spectral profile for the Brillouin gain (Stokes) and absorption (anti-Stokes) resonance, which correspond to the simulation fit in Fig. 1(d), are calculated using our model and are shown in Fig. 1(e,f), respectively. At a frequency of *ν*_*min*_ = 10.91 GHz, where the Fano resonance in Fig. 1(d) exhibits a minima, the phase difference between the Stokes and anti-Stokes field is ${\rm{\Delta }}{\varphi }_{opt}\sim$ 0.92 *π* and the amplitude of the Stokes and the anti-Stokes differ by ∼1.6 dB. Since the phase difference between the two excitation paths is nearly *π* and their amplitudes do not differ much, destructive interference between the two excitation paths is optimum at *ν*_*min*_, resulting in a minima. At frequencies greater than *ν*_*min*_, even though the amplitude difference reduces the phase difference also reduces below 0.92 *π* and the destructive interference reduces causing the RF signal to increase. For frequencies smaller than *ν*_*min*_, the amplitude difference increases whereas the phase difference approaches *π* once again resulting in reduced cancellation of the Stokes and anti-Stokes amplitudes. The coherent interaction between the Stokes and anti-Stokes excitation paths for these frequencies, therefore, leads to an increase in the RF signal. While we have demonstrated controlled excitation of the Fano resonance for *ω*_*m*_ = Ω_*B*_ in Figs 1 and 2, coherent interaction of Stokes and anti-Stokes can be achieved at any frequency to excite Fano resonance.

### Wideband excitation and control of Fano resonance

**Figure 3:**
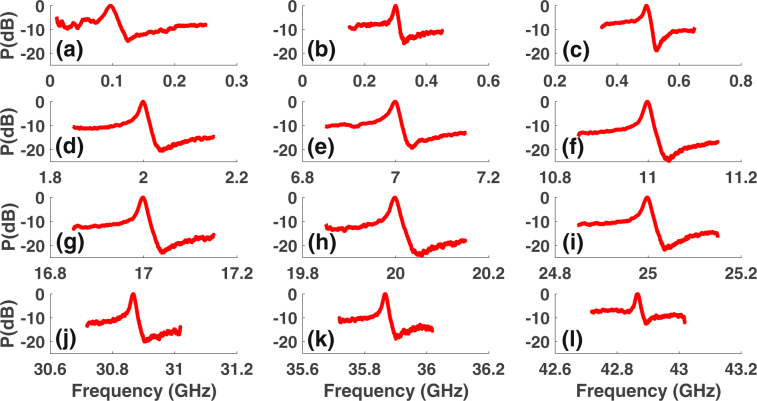
Wideband excitation of Fano resonance. (**a**–**l**) Observation of Fano resonance from 100 MHz to 43 GHz by tuning the Brillouin pump frequency. For each of the frequency, bias and other parameters were optimized to achieve large extinction.

**Figure 4:**
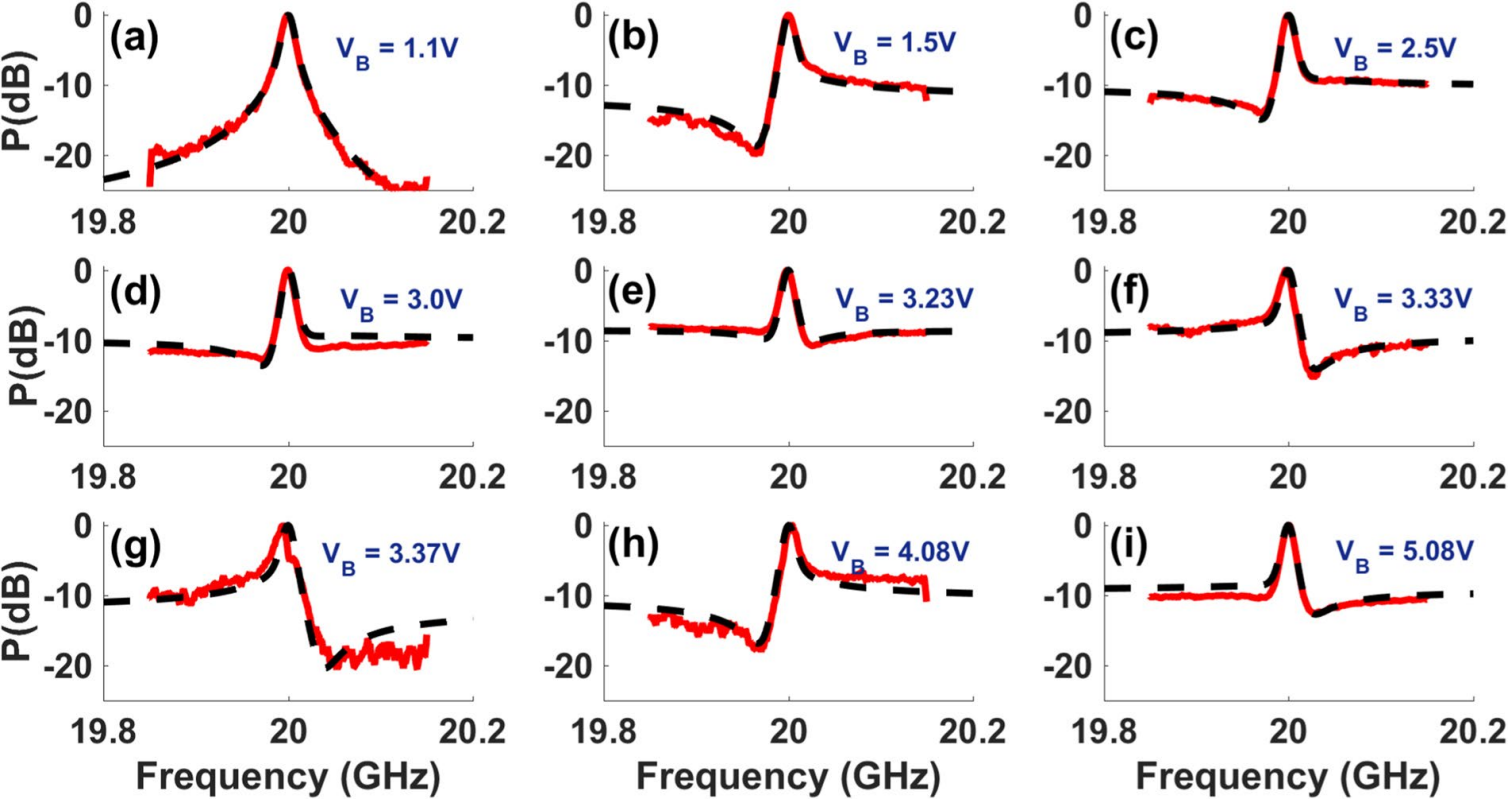
Wideband electrical control of Fano resonance. Measured (solid) Fano resonance profile at *ν*_*RF*_ = 20 GHz for different values of intensity modulator bias voltage, along with simulation fit (dashed) using Eq. 6, showing variation in extinction and polarity switching. The fit was obtained mainly by varying the value of the bias dependent parameter *α*, which demonstrates electrical control of Fano profile shape, extinction and polarity.

In order to obtain the Fano-like resonance at a frequency (*ω*_*RF*_) other than the Brillouin frequency (Ω_*B*_), we modulate the laser (*ω*_*c*_) in the pump arm with frequency (*ω*_*RF*_ + Ω_*B*_) or (*ω*_*RF*_ − Ω_*B*_) to create pumps for the Brillouin gain and loss resonance as *ω*_*RF*_ is varied up to 43 GHz. Using pump frequency tuning, we achieve controlled excitation of Fano resonance over a wide radio frequency range of 100 MHz to 43 GHz (Fig. 3(a)–(l)). By tuning the bias, we achieve electrical control of Fano resonance profile over wide frequency range. Figure 4 shows the electrical control of a Fano resonance centered at 20 GHz. Similar profiles and polarity switching were observed at other frequencies as the bias is varied. For a Z-cut intensity modulator, as the bias voltage is tuned the *α* parameter undergoes a sign change repeatedly^47^. From Fig. 4, it is evident that the Fano resonance is very sensitive to the bias voltage change and switches polarity from being left-handed minima to right-handed minima for a small change in voltage (see Fig. 4(d–i)). Polarity switching of Fano resonance has earlier been demonstrated in a plasmonic-atomic system^2^, where polarity switching occurs with a change in the angle of incidence, and other systems. Due to the sensitivity of the Fano resonance profile and depth to electrically and optically induced phase shifts, it can be used for low-power switching, sensing and high speed tuning of the RF delay at a given frequency.

The frequency of the Fano resonance in our demonstration is limited only by the frequency range of the available instruments. Coherent interaction between the Brillouin Stokes and anti-Stokes resonance results in a Fano profile when the upper and lower sidebands with the same amplitude are used. However, the Fano profile modifies to an EIT-like resonance as the ratio of the amplitude of the anti-Stokes to Stokes modulation sideband is made $\gg 1$.

### Excitation and control of induced transparency

**Figure 5:**
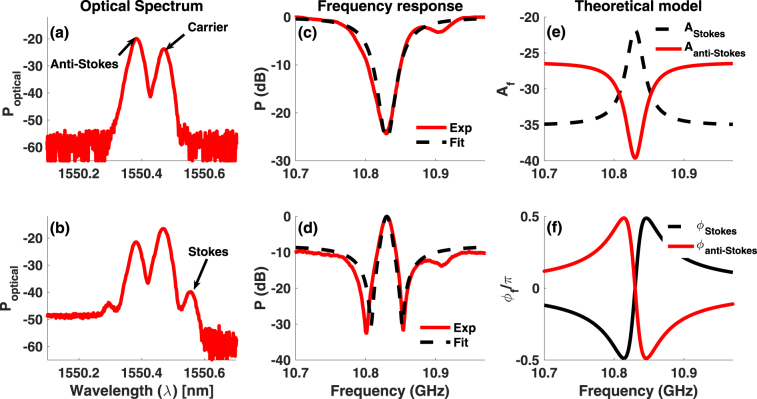
Concept of EIT-like resonance using coherent interaction of Stokes and anti-Stokes excitation pathways. (**a**) Optical spectrum, obtained after filtering the Stokes frequency using fiber Bragg gratings (FBGs) in the probe and detection path, showing a probe comprising carrier (laser) and anti-Stokes frequency. (**b**) Optical spectrum of the probe when the Stokes is allowed, by detuning the FBGs, along with the carrier and anti-Stokes. (**c**) Measured electrical frequency response (solid) of the probe in (**a**) under pump on condition showing a rejection band with a depth equal to the Brillouin loss and fit (dash) using Eq. 6 and (**d**) Measured frequency response (solid) demonstrating observation of EIT-like resonance when anti-Stokes and Stokes amplitudes are not equal, as shown in (**b**), and theoretical fit (dash). (**e**,**f**) Calculated amplitude and phase spectral response, respectively, of the Stokes (dash) and anti-Stokes (solid) corresponding to the fit in (**d**) indicating that the Stokes and anti-Stokes amplitude becomes nearly equal and phase difference approaches *π* at the rejection frequencies.

To demonstrate induced transparency, we employ the same setup as used for the excitation of Fano resonance but control the Stokes sideband amplitude using an optical filter. When the Stokes sideband is completely filtered, the probe contains only the carrier and anti-Stokes sideband (*ω*_*c*_ + *ω*_*RF*_) as shown in Fig. 5(a). The anti-Stokes sideband experiences Brillouin loss when this probe is counter-propagated with the pump. Detection of the probe using a wideband detector and VNA results in a rejection band at the Brillouin shift ${\nu }_{B}=\frac{{{\rm{\Omega }}}_{B}}{2\pi }$ (Fig. 5(c)). When the filter placed over the Stokes sideband is tuned so that some Stokes is allowed while keeping the anti-Stokes to Stokes input power ratio $\gg 1$ (Fig. 5(b)), the upper and lower sidebands experience Brillouin loss and gain resonance, respectively, as *ω*_*RF*_ is tuned around Ω_*B*_. This results in a transparency window within the rejection band when observed on the VNA (Fig. 5(d)). The depth of the transparency window is optimized by controlling the ratio of the anti-Stokes to Stokes sideband amplitude. We use Eq. (6) to obtain a fit to the induced transparency observed at Ω_*B*_. Figure 5(e,f) show the calculated amplitude and phase response, respectively, of the Stokes (dash) and anti-Stokes (solid) resonance that correspond to the fit in Fig. 5(d). At the rejection frequencies, the amplitude of the Stokes and anti-Stokes sidebands is nearly equal to within 0.1 dB (Fig. 5(e)) while their phase difference is 0.92 *π* (Fig. 5(f)), which results in destructive interference that gives rise to a larger notch depth compared to the anti-Stokes only case. The amplitude difference between the Stokes and anti-Stokes signal is ∼5 dB at the frequency for which the phase shift is *π* and therefore optimum condition of destructive interference is determined by both amplitude and phase shifts.

**Figure 6:**
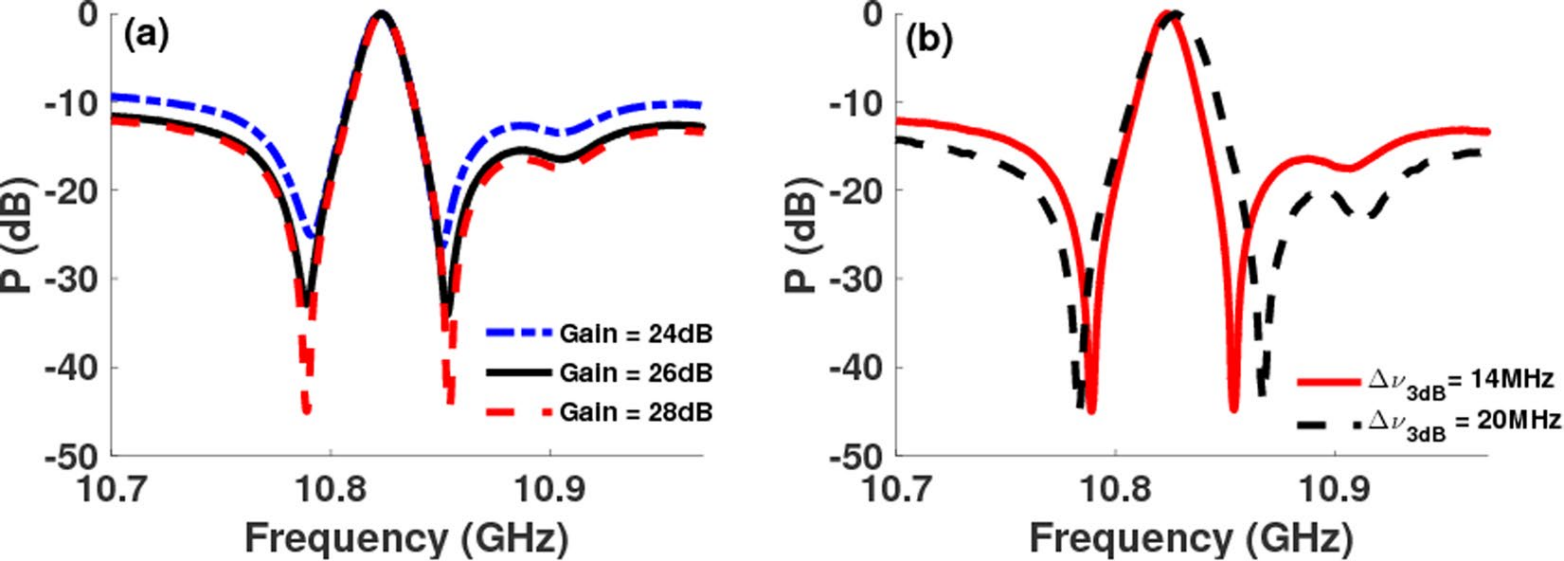
Control of induced transparency depth and linewidth. (**a**) Measured EIT-like resonance at the Brillouin shift demonstrating transparency depths of 25 dB, 35 dB, and 45 dB for Brillouin gain values of 24 dB, 26 dB, and 28 dB, respectively, with a fixed 3 dB linewidth of ∼14 MHz. (**b**) Linewidth control at a fixed transparency depth of 45 dB demonstrating 3 dB linewidths of 14 MHz and 20 MHz.

In order to demonstrate control over the depth and 3 dB linewidth of the EIT-like resonance, we tuned control parameters: Brillouin pump power, anti-Stokes to Stokes power ratio and bias to the intensity modulator. Fixing the ratio of anti-Stokes to Stokes probe amplitude and bias voltage, a small change in Brillouin gain can induce large change in transparency depth without introducing any trade-off in the 3 dB linewidth. Figure 6(a) shows that the transparency window depth varies from 25 dB (dash-dot) to 45 dB (dash) for only a 4 dB change in the Brillouin gain, while keeping the linewidth fixed at ∼14 MHz. This change in the transparency window depth happens because tuning the Brillouin gain optimizes the condition for destructive interference by making amplitudes of the Stokes and anti-Stokes signals nearly equal at frequencies where the phase difference between them approaches *π*. At a fixed Brillouin pump power and bias, controlling the ratio of the anti-Stokes to Stokes amplitude also optimizes the transparency depth. Figure 6(b) shows the control of 3 dB linewidth, which was tuned from 14 MHz to 20 MHz by varying the control parameters, at a fixed transparency depth of 45 dB. Coherent interaction of the Brillouin Stokes and anti-Stokes excitation pathways, therefore, provides a highly flexible approach to realize EIT-like resonance where the linewidth is increased by nearly 43% at a fixed transparency depth and large tuning of transparency depth is demonstrated without compromising the linewidth for only a small change in Brillouin gain.

**Figure 7:**
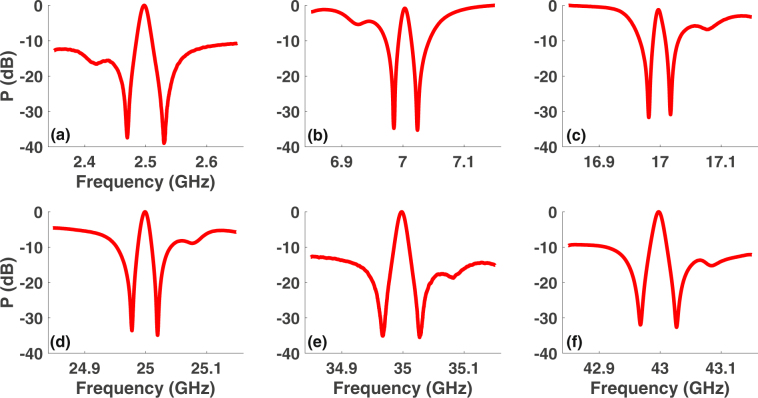
Observation of wideband induced transparency. EIT-like resonance over a microwave frequency range of 2.5 GHz to 43 GHz (**a**–**f**) while maintaining a depth of >30 dB for the transparency window and keeping the 3 dB linewidth fixed at 12 ± 1.5 MHz.

Next we demonstrate induced transparency over a record frequency range of 2.5 GHz–43 GHz (Fig. 7(a–f)) by tuning the Brillouin pump frequency using intensity modulation, as in the case of wideband Fano, and varying our control parameters. The range can be extended further to higher frequencies using intensity modulators with higher bandwidths. The depth and 3 dB linewidth of the induced transparency is controlled for each frequency by tuning the bias, pump power, and the ratio of the anti-Stokes to Stokes power to achieve a depth of >30 dB and 3 dB linewidth of 12 ± 1.5 MHz. At the frequency of 43 GHz, we observe a transparency window with an ultra narrow bandwidth ∼13 MHz, which gives an extremely high Q-factor of 3300. A minimum 3 dB linewidth ∼10 MHz is observed at 17 GHz. The excitation and control of Fano resonance and induced transparency exploiting coherent interaction of Brillouin Stokes and anti-Stokes resonance only requires control of the Brillouin pump power, anti-Stoke to Stokes power ratio, and voltage bias and is not dependent on coupling between different mechanical resonators nor needs precise control over the device geometry.

## Discussion

We have demonstrated wideband excitation and control, using optical and electrical means, of Fano and EIT-like resonance exploiting coherent interaction of Brillouin Stokes and anti-Stokes resonances. For Fano profile, this interaction makes the extinction, polarity and the frequency of minimum spectral amplitude highly sensitive to optical pump power and bias voltage. Although Fano and EIT-like resonances have been demonstrated earlier at optical, terahertz and microwave frequency regimes, wideband excitation of both the phenomena in a controlled manner had been challenging because it relies upon wideband tuning of the atomic or structural resonance and their precise control and coupling. The high flexibility of the SBS process that originates from the dependence of the frequency, profile, and bandwidth of the Brillouin gain (loss) spectrum on Brillouin pump parameters, makes it a suitable system to study wideband excitation and control of Fano and induced transparency.

Unlike EIT in atomic systems, where destructive interference between two transition amplitudes introduces transmission in a narrow spectral region, the destructive interference between the Stokes and anti-Stokes amplitudes creates symmetrically placed rejection bands with large depth that amplifies the transparency amplitude introduced due to the Brillouin gain resonance. The depth of these rejection bands and 3 dB linewidth of the transparency window are highly sensitive to ratio of the anti-Stokes to Stokes signal amplitude and pump power. By controlling this ratio and Brillouin gain, large change (>20 dB) in the transparency depth is achievable with a small change in the gain without affecting the linewidth. Tuning of anti-Stokes to Stokes amplitude ratio and gain parameter allows control of the linewidth at fixed transparency depth. In this demonstration, induced transparency with unprecedented 3 dB linewidth ∼10 MHz is observed at 17 GHz, which can be further reduced by controlling the anti-Stokes to Stokes amplitude ratio and Brillouin pump power.

In order to understand the Fano and EIT-like resonance using coherent interaction of Brillouin pathways, we developed a model that accurately predicts the frequency response for different pump powers, bias values and anti-Stokes to Stokes power ratio. The model gives an understanding of the amplitude and phase conditions required to control the shape, polarity, extinction, and linewidth of Fano and EIT-like resonances.

Demonstration of Fano resonance and induced transparency using SBS and their control over a wide frequency range with electrical and optical means offers potential for applications in low-power switching and microwave photonic signal processing. Intensity modulators^49^, SBS^50^ and other components used in our work have, recently, been demonstrated on optical chip which opens up the development of photonic integrated circuit devices^15,16^ for controlled wideband excitation of Fano and EIT-like resonance and their application to low-power switching, microwave photonic signal processing, and sensing.

## Methods

### Fano resonance

**Figure 8:**
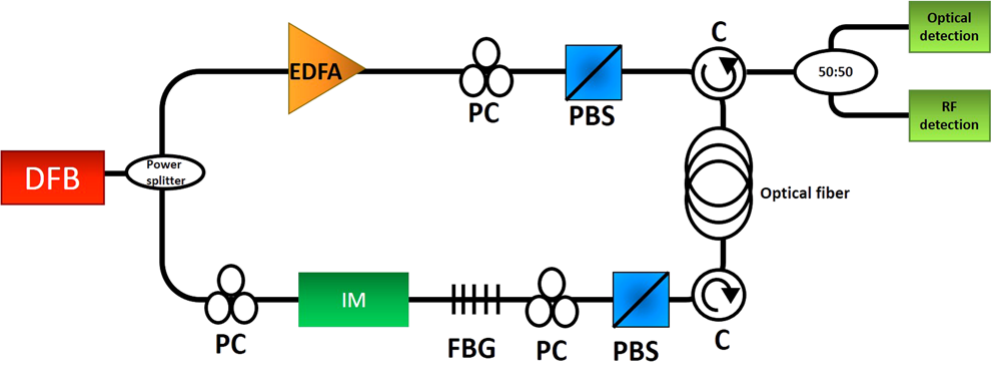
Experimental setup for the observation of Fano resonance and induced transparency: DFB- distributed feedback laser; EDFA- Erbium-doped fiber amplifier; PC- polarisation controller; IM- intensity modulator driven by an RF source and DC power supply; FBG- fiber Bragg grating; PBS- polarization beam splitter; C- circulators; Optical detection (Optical spectrum analyzer); RF detection (wideband photodetector and vector network analyzer).

To demonstrate Fano resonance, we set up the pump-probe experiment(Fig. 8) that is used to characterize gain profile in backward SBS process. A continuous wave distributed feedback laser (DFB: Teraxion Inc.) emitting light at 1550 nm is split using power splitter to generate signals for the pump and probe arm. In the pump arm, a high power (2 W) erbium-doped fiber amplifier (HP-EDFA: Amonics) is used to control the pump power. In the probe arm, an intensity modulator, driven by a vector network analyser (VNA: Keysight) and a DC power supply (Hameg), is used to generate the Stokes and anti-Stokes sidebands. The pump and probe signals are then launched into a 50 m long polarization maintaining (PM) fiber in the counter-propagating direction through fiber polarisation controllers (FPCs), polarization beam splitters (PBSs) and PM circulators. The output probe signal is split using a 3 dB power splitter and recorded using an optical spectrum analyser (OSA: Anritsu) and a VNA connected to a wide bandwidth photodetector (Finisar). In order to observe the Fano feature at the Brillouin shift (Ω_*B*_), frequency of the VNA is scanned around Ω_*B*_.

In order to demonstrate Fano resonance at frequencies (*ω*_*RF*_) other than Ω_*B*_, the laser output is amplified using an EDFA (PriTel) before splitting it into the pump and the probe arm. Carrier suppressed double sideband modulation (CS-DSB) is used in the pump arm to tune the pump frequency. The carrier in the pump arm is suppressed by adjusting the bias to an intensity modulator and using a fiber Bragg grating (FBG) before the HP-EDFA (Amonics). The modulation frequency in the pump arm is kept at *ω*_*RF*_ + Ω_*B*_ or *ω*_*RF*_ − Ω_*B*_ using an RF signal generator (Keysight) whereas probe is generated by modulating the laser (carrier) at frequency *ω*_*RF*_ with a VNA. Pump and probe are then counter-propagated in a 50 m long PM fiber through FPCs, PBSs and PM circulators. The probe power is controlled using a low-noise EDFA (Pritel) for different experiments. Optical and electrical response of probe is then recorded using OSA, photodetector and VNA.

### Induced transparency

To demonstrate EIT-like resonance, a 500 m long single mode fiber (SMF) is used as the Brillouin medium. The setup utilised for observing induced transparency is similar to that of Fano. Unlike Fano, where equal amplitude Stokes and anti-Stokes sidebands are used, observation of induced transparency requires control of the anti-Stokes to Stokes power ratio. Fiber Bragg gratings (FBGs) are used to control Stokes power in the probe arm. To obtain the loss profile in Fig. 5(c), an additional FBG is added at the output port to allow only the carrier and anti-Stokes sideband to propagate to the OSA and VNA. FPCs are used to align the polarization of pump and probe before launching them in counter-propagating direction, through circulators, into an SMF.

The setup for EIT at frequencies other than Ω_*B*_ utilises the similar modified approach as used in wideband Fano. The laser output is fed into a high power erbium-doped fiber amplifier (HP-EDFA: PriTel) and split using a 50:50 splitter. One arm of the splitter is used to generate the pump using CS-DSB modulation. The other arm generates the probe and FBGs are utilised in this path to suppress the sideband that would see gain. The ratio of Stokes and anti-Stokes is tuned by tuning the FBGs to obtain the required transparency depth and the probe signal is recorded on OSA and VNA.

### Data availability

Data sets and simulation codes generated during the current study are available from the corresponding author on a reasonable request.
